# Identification *BCL6* and miR-30 family associating with Ibrutinib resistance in activated B-cell-like diffuse large B-cell lymphoma

**DOI:** 10.1007/s12032-021-01470-5

**Published:** 2021-02-25

**Authors:** Jiazheng Li, Yan Huang, Yun Zhang, Jingjing Wen, Yanxin Chen, Lingyan Wang, Peifang Jiang, Jianda Hu

**Affiliations:** Fujian Provincial Key Laboratory of Hematology, Fujian Institute of Hematology, Fujian Medical University Union Hospital, 29 Xinquan Road, Fuzhou, 350001 China

**Keywords:** Activated B-cell-like diffuse large B cell lymphoma, Ibrutinib resistance, Bioinformatic analysis, *BCL6*, miR-30 family

## Abstract

Ibrutinib has clear efficacy for activated B-cell-like diffuse large B cell lymphoma (ABC-DLBCL) in previous clinical researches. However, the resistance of Ibrutinib has limited its therapeutic benefit and the potential mechanism remains unclear. This study was aimed to identify potential candidate genes and miRNA targets to overcome Ibrutinib resistance in ABC-DLBCL. First, two expression profiles were downloaded from the GEO database, which used to identify the DEGs related to Ibrutinib resistance in ABC-DLBCL cell lines by GEO2R analysis separately. And the common DEGs were obtained though Venn diagram. Then Gene ontology (GO) and pathway enrichment analysis were conducted by DAVID database. From STRING database, *BCL6*, *IL10*, *IL2RB*, *IRF4*, *CD80*, *PRDM1*and *GZMB* were determined to be the hub genes by protein–protein interaction (PPI) network. Through miRNA-mRNA targeting network, we found that *BCL6*, *IRF4*, *CD80*, and *PRDM1* were common target genes of miR-30 family. The cBioPortal database showed that *BCL6* had the highest level of genetic alterations among DLBCL. In addition, another expression profile from GEO database showed that *BCL6* was significantly high expression in no responsive patients after Ibrutinib treatment, and the receiver operating characteristic (ROC) curve which was used to evaluate the relationship between *BCL6* expression and its effect was 0.67. MTT assay showed that treatment with FX1 (a *BCL6* inhibitor) can enhance the sensitivity of Ibrutinib in C481S BTK HBL-1 cells. The results suggested that *BCL6* and miR-30 family maybe associate with Ibrutinib resistance in ABC-DLBCL.

## Introduction

Diffuse large B cell lymphoma (DLBCL) were classified as germinal center B-cell-like (GCB) and activated B-cell-like (ABC) DLBCL with cell-of-origin (COO) [1, 2]. And the ABC subtype presents poor prognosis [3]. Although targeted therapy drugs improved the prognosis of DLBCL patients. However, about 40% patients of DLBCL could not benefit from first-line therapy, and drug resistance is a leading cause of it [4].

Drug resistance relates to various mechanisms, such as gene-driven, pathway mediated. *TBL1XR1*, *IRF4*, *TP53*, *FOXO1*, *KMT2C (MLL3)*, *CCND3*, *NFKBIZ,* and *STAT6*, were potential candidate targets to overcome drug resistance in DLBCL [5, 6]. In addition, increasing evidences have revealed miRNAs negatively regulated expression of their target genes and abnormally expressed in many tumors, including DLBCL [7, 8]. A meta-analysis suggests that DLBCL patients with abnormal expression of miR-155, miR-17/92 clusters, miR-21, miR-224, or miR-146b-5p are associated with worse outcome and higher risk of drug resistance [9]. Currently, more and more DLBCL related genes and signaling pathways have been identified, and a number of targeted therapeutic drugs have recently been introduced, such as Ibrutinib.

Ibrutinib, a small molecule inhibitor of Brutons tyrosine kinase (BTK), was approved to use for several B-cell malignancies by the United States Food and Drug Administration (FDA) in 2013 [10]. The curative effect of Ibrutinib monotherapy on rel/ref ABC DLBCL is significantly better than that of GCB subtype [11]. However, resistance to ibrutinib limits its effectiveness, and the underling mechanisms are still not clear. Bioinformatics analysis is used to analyze data of high-throughput sequencing, which helps us to study potential molecular mechanisms of drug resistance. The present study generated differentially expressed genes (DEGs) to identified the core gene among their regulatory relations and miRNA targets associated with Ibrutinib resistance in ABC-DLBCL.

## Materials and methods

### Microarray data

The three microarray expression profile datasets (GSE138126、GSE93984 and GSE93985) were obtained from the gene expression omnibus (GEO, http://www.ncbi.nlm.nih.gov/geo/) database [12]. GSE138126 was determined by GPL13497 Agilent-026652 Whole Human Genome Microarray 4 × 44 K (Submission date: Sep 29, 2019, Last update date: Nov 03, 2019), a total of 6 Ibrutinib-resistant and 6 sensitive samples, including HBL-1 and OCI-LY10 ABC DLBCL cell lines. And GSE93985 was based on the comparison between sensitive TMD8 ABC DLBCL and its Ibrutinib-resistant cell lines. It was performed by GPL17586.0 Affymetrix Human Transcriptome Array 2.0 (Submission date: Jan 24, 2017, Last update date: Oct 29, 2018). The GSE93984 was detected by GPL570 U133 plus 2.0 arrays, which included Ibrutinib pretreated tumor biopsy samples from ABC-DLBCL patients.

### DEGs screening

The DEGs between Ibrutinib sensitive and resistant cell lines were obtained by GEO2R analysis with cutoff values of *P* value < 0.05 and |Log_2_FC|> 1 [13], a web application based on R software in GEO database. Then we used limma package of R software to constructed Volcano maps showing the DEGs and Draw Venn Diagram website (http://bioinformatics.psb.ugent.be/webtools/Venn/) to get the overlapped DEGs. The overlapped DEGs were considered to be associated with Ibrutinib resistance.

### Gene ontology (GO) and pathway analysis

GO analysis was carried out to analyze different functions of Ibrutinib resistance-related DEGs including biological process (BP), cellular component (CC), molecular function (MF) category. Pathway enrichment analysis was carried out with Kyoto Encyclopedia of Genes and Genomes (KEGG) and Reactome database. In this work, GO terms and pathway analysis were both performed by Annotation, Visualization and Integrated Discovery (DAVID, http://david.abcc.ncifcrf.gov/) (vision 6.8) [14] database with *P* value < 0.05 as the threshold value.

### Integration of protein–protein interaction (PPI) network

Search Tool for the Retrieval of Interacting Genes (STRING, http://string-db.org) (vision 11.0) [15] database was used to generate PPI network to show the association among DEGs. Further, PPI network was visualized through cytoscape (vision 3.7.2) software [16], then the top DEGs with Maximal Clique Centrality (MCC) score > 10,000 were regarded as hub genes by CytoHubba plugin.

### Exploring genetic alterations of hub genes and predicting hub gene-related miRNAs

The cBioPortal database (http://www.cbioportal.org/) [17] was used to explore genetic alterations of hub genes. The miRNAs who target hub genes were predicted by miRDB database (http://mirwalk.umm.uni-heidelberg.de) [18] and the miRNA–mRNA interaction was constructed by Cytoscape (vision 3.7.2) software.

### *BCL6* expression in ABC-DLBCL patients with different outcome after Ibrutinib treatment

In the GSE93984 expression profile, 17 ABC-DLBCL patients were classified into no responsive (stable disease + progression disease, SD + PD) group, and 11 ABC-DLBCL patients were responsive (complete response + partial response, CR + PR) group after Ibrutinib treatment. We used the GraphPad Prism Software 7.0 to visualize the relative expression of *BCL6* between above two groups, and the ROC R package was performed to operate ROC curves.

### Cell culture

C481S BTK HBL-1 cells, the BTK C481S mutant induced resistance to Ibrutinib, were a gift from Prof. Zhu J (Key laboratory of Carcinogenesis and Translational Research, Department of Lymphoma, Peking University Cancer Hospital & Institute). C481S BTK HBL-1 cells were cultured in RPMI-1640 medium (BasalMedia, China), 10% fetal bovine serum (FBS, Gibco, UT) and 0.05 mM 2-mercaptoethanol (Sigma, USA) at 37 °C with 5% CO_2_ with in a humidified incubator (Thermo, USA).

### Ibrutinib sensitivity assay

The effect of Ibrutinib or combination with FX1 (a *BCL6* inhibitor) on C481S BTK HBL-1 cells was detected by 3-(4,5-dimethylthiazol2-yl)-2,5 diphenyltetra-zolium bromide (MTT, Sigma, MO, USA) assay in vitro. C481S BTK HBL-1 cells were seeded at a density of 4 × 10^4^/well and incubated with different concentrations of Ibrutinib (0, 0.5, 1, 2, 4, and 8 µM) with/without 17.5 µM FX1 at 37 °C in a 5% CO_2_ incubator for 48 h. Then cells were incubated with 20 μL MTT (5 mg/mL) in the last four hours of the experiment. Optical density (OD) value was measured at the wave length of 490 nm and 630 nm by an Elx808 Absorbance Microplate spectrophotometer (BioTek, UT).

### Statistics analysis

Each experiment was performed in triplicate. Measurement data were expressed as mean ± standard deviations and unpaired *t* test, processed by SPSS statistics software 25.0 or GraphPad Prism Software 7.0, where *P* < 0.05 means significant statistical differences.

## Results

### Identification of DEGs between Ibrutinib sensitive and resistant cell lines in ABC-DLBCL

**Fig. 1:**
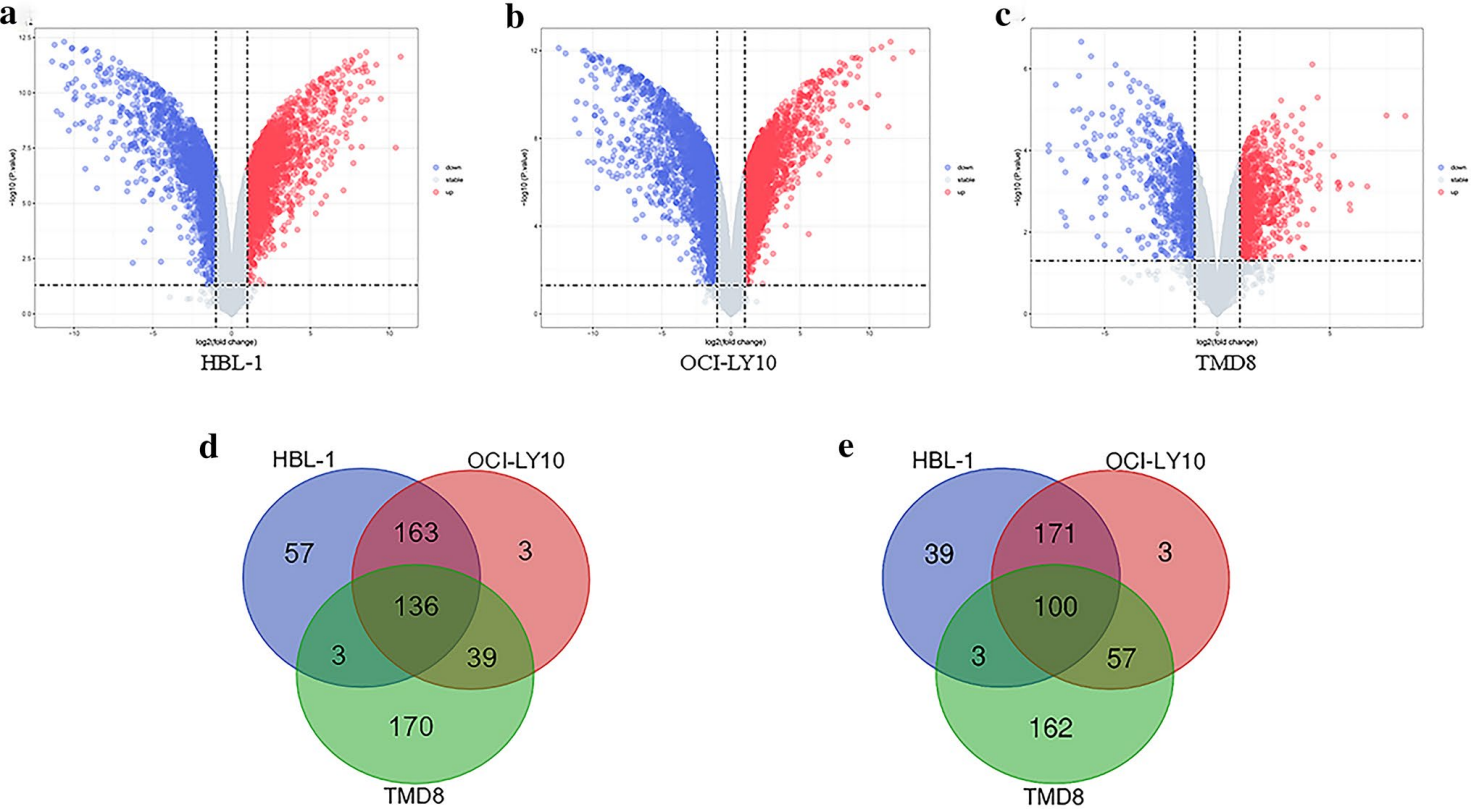
Identification of DEGs between Ibrutinib-resistant cell lines and sensitive cell lines in ABC-DLBCL. Volcano plot of **a** HBL-1, **b** OCI-LY10 and **c** TMD8. Blue plots represent downregulated genes, while red plots represent upregulated genes. Identification of the oncordant expression changes **d** downregulated **e** upregulated DEGs from three cell lines were performed by Draw Venn Diagram website (http://bioinformatics.psb.ugent.be/webtools/Venn/). Blue circles indicate HBL-1 cell line, red circles indicate OCI-LY10 cell line, green circles indicate TMD8 cell line

The two mRNA expression profiles, GSE138126 and GSE93985, were obtained from the GEO database including 3 ABC-DLBCL cell lines (HBL-1, OCI-LY10, TMD8), as shown in Table 1. We obtained 6191, 7063, 2531 DEGs from above 3 cell lines, respectively, with *P* < 0.05 and |Log_2_FC|≥ 1 as cutoff values by GEO2R analysis. Volcano plot of the DEGs between Ibrutinib sensitive and resistant cell lines were generated (Fig. 1a–c). A total of 671 common DEGs were identified from above 3 cell lines, and 236 of 671 common DEGs showed concordant expression changes, among them, 136 downregulated and 100 upregulated (Fig. 1d, e). Therefore, above 236 common DEGs would be used for subsequent analysis.

**Table 1:** Information of the two microarray expression profiles

GEO ID	Platform	Cell line	Samples
GSE138126	GPL13497 Agilent-026652 Whole Human Genome Microarray 4 × 44 K	HBL-1	3 Ibrutinib-resistant/3 parental cell lines
GSE138126	GPL13497 Agilent-026652 Whole Human Genome Microarray 4 × 44 K	OCI-LY10	3 Ibrutinib-resistant/3 parental cell lines
GSE93985	GPL17586[HTA-2_0] Affymetrix Human Transcriptome Array 2.0	TMD8	2 Ibrutinib-resistant/2 parental cell lines

### GO analysis and pathway analysis

**Fig. 2:**
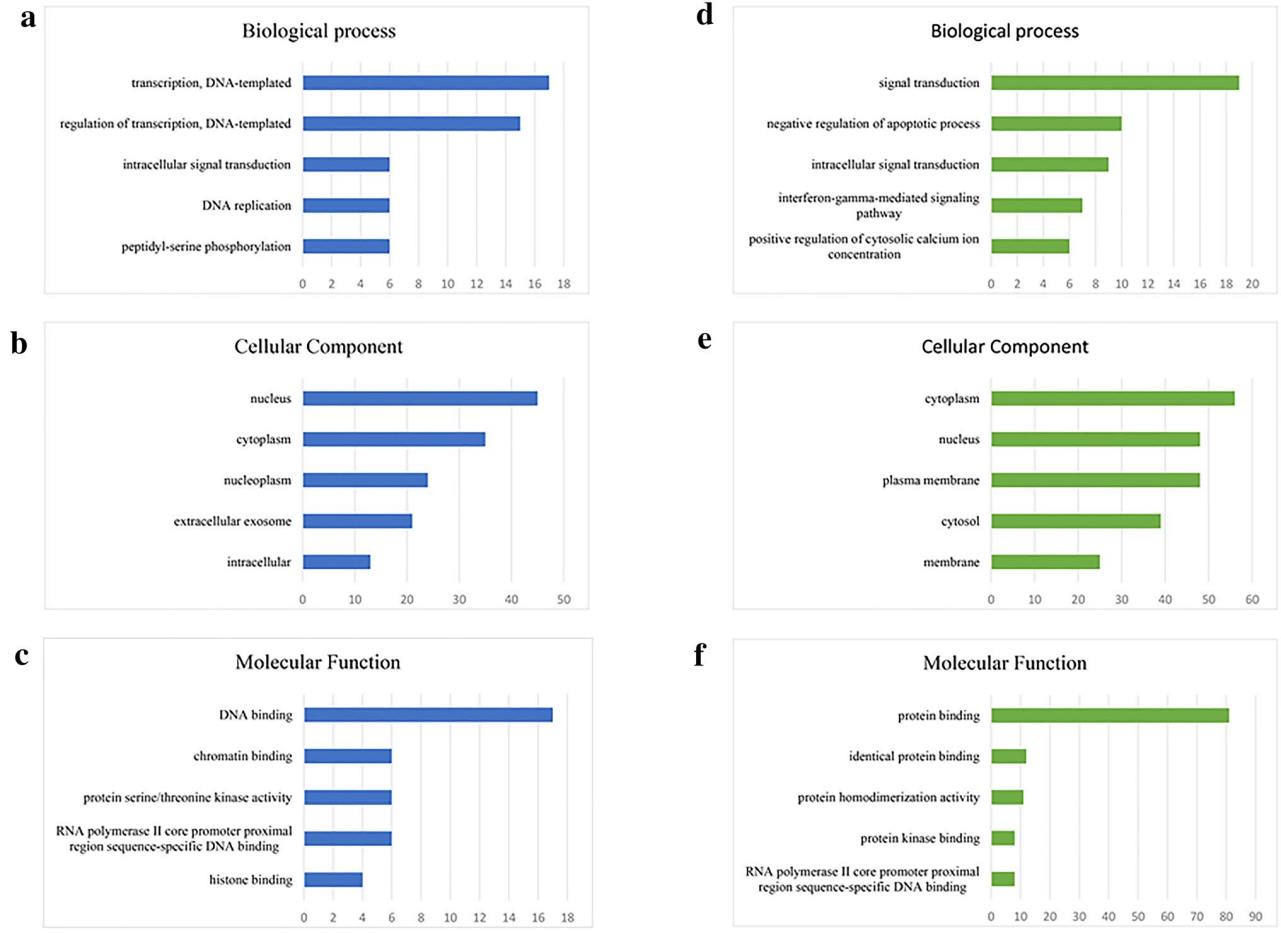
GO analysis of DEGs. Enrichment of **a** biological process, **b** cellular components, and **c** molecular function for upregulated DEGs. Enrichment of **d** biological process, **e** cellular components, and **f** molecular function for downregulated DEGs. DEGs functional enrichment was analyzed using GO analysis on DAVID database (vision 6.8)

We performed GO analysis to analyze the functional role of resistance-related DEGs using DAVID database (vision 6.8). The top 5 GO terms were selected to display according to count (numbers of related gene). Biological process indicated that upregulated DEGs were correlated with transcription (Fig. 2a) while downregulated DEGs were signal transduction and negative regulation of apoptotic process (Fig. 2d). And both the enrichment in cellular components mainly related to nucleus and cytoplasm (Fig. 2b, e). Under the category of molecular function, upregulated DEGs were significantly enriched in DNA binding and chromatin binding (Fig. 2c), whereas downregulated DEGs were mainly enriched in protein binding (Fig. 2f).

KEGG Pathway and Reactome database were performed to learn Ibrutinib resistance-related signaling pathway in ABC-DLBCL. The Table 2 and Table 3 listed the downregulated and upregulated DEGs significantly enriched pathway (*P* < 0.05), respectively. The common signaling pathway was cell cycle pathway in upregulated DEGs while cytokines-based signaling pathways in downregulated DEGs.

**Table 2:** The significant pathway enrichment analysis of upregulated DEGs

Category	Term	*P* value	Genes
KEGG	hsa04110: cell cycle	1.78E−02	HDAC1, CDKN2C, CDC25A, ATM
KEGG	hsa04910: insulin signaling pathway	2.36E−02	PPP1R3E, PRKAR2A, PRKCI, PIK3R3
KEGG	hsa04390: hippo signaling pathway	2.97E−02	PPP2R1B, TEAD4, PRKCI, LEF1
KEGG	hsa05202: transcriptional misregulation in cancer	3.84E−02	HDAC1, CDKN2C, BCL6, ATM
REACTOME	R-HSA-113510: E2F mediated regulation of DNA replication	2.85E−03	DHFR, RRM2, CDC25A
REACTOME	R-HSA-69205: G1/S-specific transcription	2.85E−03	DHFR, RRM2, CDC25A
REACTOME	R-HSA-1538133: G0 and early G1	6.14E−03	RBBP4, HDAC1, CDC25A

**Table 3:** The significant pathway enrichment analysis of downregulated DEGs

Pathway	Term	*P* value	Genes
KEGG	hsa05330: allograft rejection	4.19E−05	CD80, GZMB, HLA-DPA1, HLA-DPB1, HLA-DOA, IL10
KEGG	hsa04672: intestinal immune network for IgA production	1.36E−04	CD80, CCR10, HLA-DPA1, HLA-DPB1, HLA-DOA, IL10
KEGG	hsa05145: toxoplasmosis	1.64E−04	CIITA, HLA-DPA1, JAK2, HLA-DPB1, HLA-DOA, IL10, AKT3, STAT3
KEGG	hsa05320: autoimmune thyroid disease	2.20E−04	CD80, GZMB, HLA-DPA1, HLA-DPB1, HLA-DOA, IL10
KEGG	hsa05332: graft-versus-host disease	3.99E−04	CD80, GZMB, HLA-DPA1, HLA-DPB1, HLA-DOA
KEGG	hsa04940: type I diabetes mellitus	1.01E−03	CD80, GZMB, HLA-DPA1, HLA-DPB1, HLA-DOA
KEGG	hsa05152: tuberculosis	2.79E−03	CIITA, HLA-DPA1, JAK2, HLA-DPB1, HLA-DOA, IL10, AKT3, CD74
KEGG	hsa05416: viral myocarditis	3.17E−03	CD55, CD80, HLA-DPA1, HLA-DPB1, HLA-DOA
KEGG	hsa05310: asthma	3.94E−03	HLA-DPA1, HLA-DPB1, HLA-DOA, IL10
KEGG	hsa05321: inflammatory bowel disease (IBD)	4.82E−03	HLA-DPA1, HLA-DPB1, HLA-DOA, IL10, STAT3
KEGG	hsa04920: adipocytokine signaling pathway	6.62E−03	CD36, SOCS3, JAK2, AKT3, STAT3
KEGG	hsa05140: leishmaniasis	6.96E−03	HLA-DPA1, JAK2, HLA-DPB1, HLA-DOA, IL10
KEGG	hsa04612: antigen processing and presentation	8.83E−03	CIITA, HLA-DPA1, HLA-DPB1, HLA-DOA, CD74
KEGG	hsa05164: influenza A	1.08E−02	CIITA, SOCS3, HLA-DPA1, JAK2, HLA-DPB1, HLA-DOA, AKT3
KEGG	hsa04978: mineral absorption	1.15E−02	HMOX1, MT2A, STEAP1, MT1F
KEGG	hsa04062: chemokine signaling pathway	1.46E−02	LYN, PREX1, HCK, CCR10, JAK2, AKT3, STAT3
KEGG	hsa04630: jak-STAT signaling pathway	1.95E−02	IL2RB, SOCS3, JAK2, IL10, AKT3, STAT3
KEGG	hsa05150: *staphylococcus aureus* infection	2.00E−02	HLA-DPA1, HLA-DPB1, HLA-DOA, IL10
KEGG	hsa04145: phagosome	2.23E−02	CD36, TFRC, TUBB6, HLA-DPA1, HLA-DPB1, HLA-DOA
KEGG	hsa04931: insulin resistance	2.86E−02	CD36, SOCS3, PTPN1, AKT3, STAT3
KEGG	hsa04917: prolactin signaling pathway	4.06E−02	SOCS3, JAK2, AKT3, STAT3
KEGG	hsa05169: epstein-Barr virus infection	4.19E−02	LYN, HLA-DPA1, HLA-DPB1, AKT3, STAT3
KEGG	hsa05168: herpes simplex infection	4.65E−02	SOCS3, HLA-DPA1, JAK2, HLA-DPB1, HLA-DOA, CD74
REACTOME	R-HSA-877300: interferon gamma signaling	1.34E−05	CIITA, TRIM2, SOCS3, MT2A, HLA-DPA1, JAK2, IRF4, HLA-DPB1
REACTOME	R-HSA-982772: growth hormone receptor signaling	5.73E−05	LYN, SOCS3, JAK2, PTPN1, STAT3
REACTOME	R-HSA-2132295: MHC class II antigen presentation	7.94E−04	EHHADH, CTSO, TUBB6, HLA-DPA1, HLA-DPB1, HLA-DOA, CD74
REACTOME	R-HSA-389513: CTLA4 inhibitory signaling	9.67E−04	LYN, CD80, PPP2R5C, AKT3
REACTOME	R-HSA-114604: GPVI-mediated activation cascade	1.59E−03	IL2RB, GAB2, LYN, JAK2, AKT3
REACTOME	R-HSA-2586552: signaling by Leptin	4.21E−03	SOCS3, JAK2, STAT3
REACTOME	R-HSA-1059683: interleukin-6 signaling	4.21E−03	SOCS3, JAK2, STAT3
REACTOME	R-HSA-1433557: signaling by SCF-KIT	4.43E−03	GAB2, LYN, JAK2, STAT3
REACTOME	R-HSA-877312: regulation of IFNG signaling	6.84E−03	SOCS3, JAK2, PTPN1
REACTOME	R-HSA-392451: G beta:gamma signaling through PI3Kgamma	9.18E−03	IL2RB, GAB2, JAK2, AKT3
REACTOME	R-HSA-912526: interleukin receptor SHC signaling	2.45E−02	IL2RB, GAB2, JAK2
REACTOME	R-HSA-202433: generation of second messenger molecules	4.17E−02	ENAH, HLA-DPA1, HLA-DPB1

### PPI network and hub genes identification

**Fig. 3:**
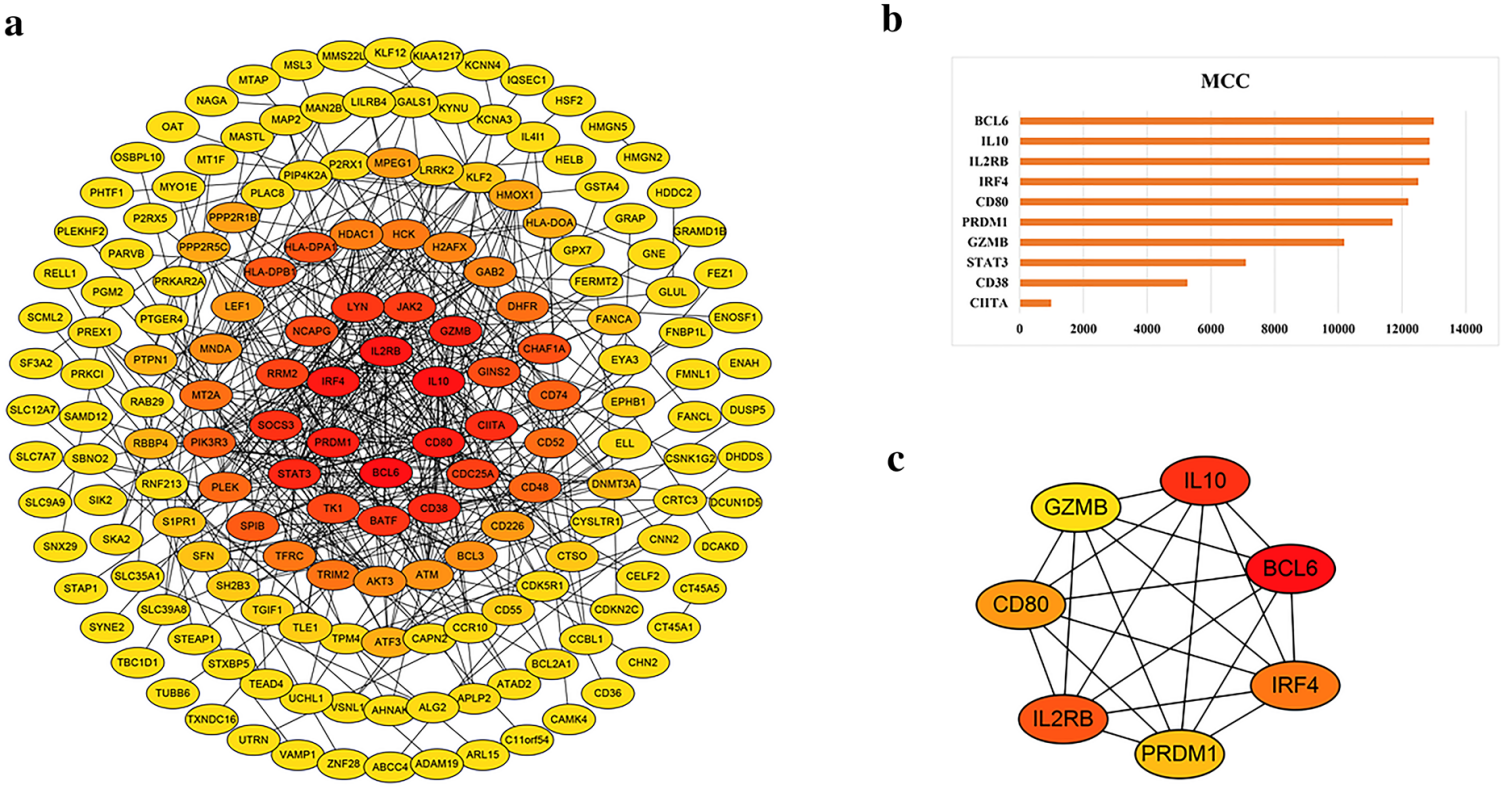
The PPI network and hub genes identification. **a** The PPI network of DEGs was visualized by Cytoscape software. **b** The top 10 DEGs based on MCC algorithm analysis. **c** The hub genes were identified with the MCC score cutoff of 10,000

String database and Cytoscape (vision 3.7.2) software were used to study and visualize the correlation among the DEGs at the protein level. The Ibrutinib resistance-related PPI network included 228 nodes and 430 edges with PPI enrichment (*P* value < 1.0e−16) (Fig. 3a). The top 10 DEGs based on MCC score in above PPI network were identified according to cytoHubba plugin in Cytoscape software as in Fig. 3b. With the threshold of MCC score > 10,000, a total of seven genes were considered hub genes: *BCL6*, *IL10*, *IL2RB*, *IRF4*, *CD80*, *PRDM1*, *GZMB* (Fig. 3c). And *BCL6* got the highest score so that *BCL6* may be the most important gene among them.

### Construction of miRNA-mRNA interaction

**Fig. 4:**
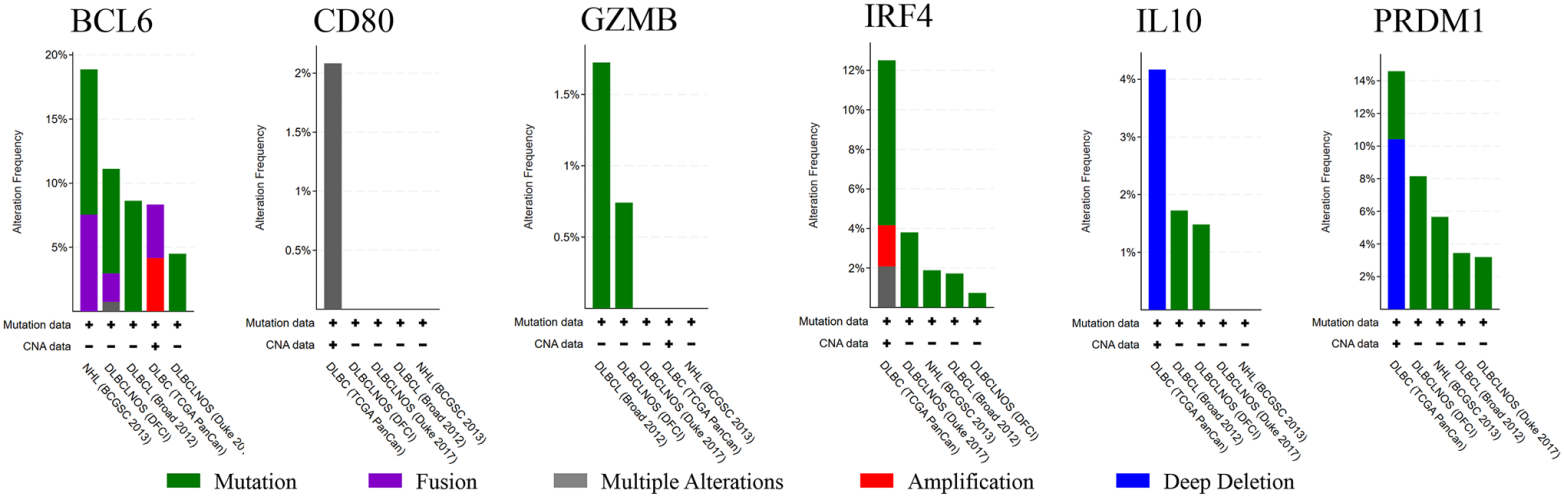
The histogram of the genetic alteration frequencies of hub genes across five DLBCL datasets (DFCI, Nat Med 2018, BCGSC, Blood 2013, Broad, PNAS 2012, Duke, Cell 2017, TCGA, PanCancer Atlas)

MiRDB database was used to analyze miRNA associated with hub genes which might affect Ibrutinib resistance in DLBCL. Every predicted miRNA in miRDB database has a prediction score so that we defined > 70 as the threshold level. However, we could not find miRNA that targets *GZMB*. Next, Cytoscape software was used to generate miRNA–mRNA interaction among 6 hub genes (Fig. 4). We found that hsa-miR-30a-5p, hsa-miR-30b-5p, hsa-miR-30c-5p, hsa-miR-30d-5p, and hsa-miR-30e-5p targeted for *BCL6*, *PMDR1*, *CD80,* and *IRF4* simultaneously. And above miRNAs belong to miRNA-30 family members.

### Genetic alteration of hub genes

**Fig. 5:**
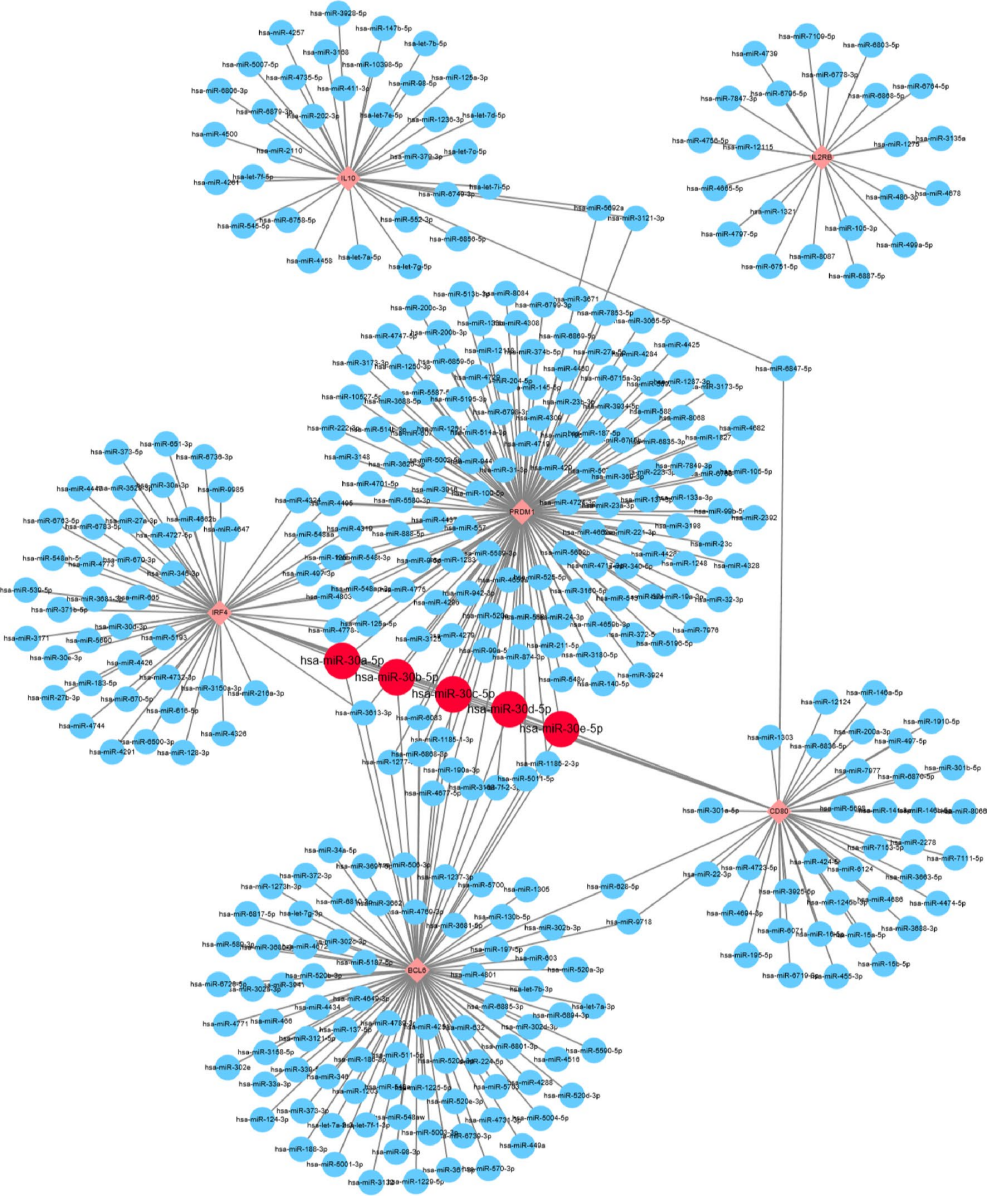
The construction of miRNA–mRNA interaction of hub genes in ABC-DLBCL. Diamond represents hub gene and circle represents predicted the potential miRNA

We used cBioportal database to explore the genetic alterations of hub genes including 1295 samples in five DLBCL datasets (DFCI, Nat Med 2018, BCGSC, Blood 2013, Broad, PNAS 2012, Duke, Cell 2017, TCGA, PanCancer Atlas) (Fig. 5). Genetic alteration of *IL2RB* was not found while genetic alterations of the remaining six hub genes included mutation, fusion, multiple alterations, amplification, and deep deletion. Mutation was the most common genetic alterations among these hub genes. Significantly, we found that *BCL6* showed the highest level of genetic alterations among hub genes. According to the result of PPI network and genetic alterations, we thought the *BCL6* may play the most important role among these hub genes. Therefore, we validated the *BCL6* in following analysis and experiment.

### Validation of *BCL6*

**Fig. 6:**
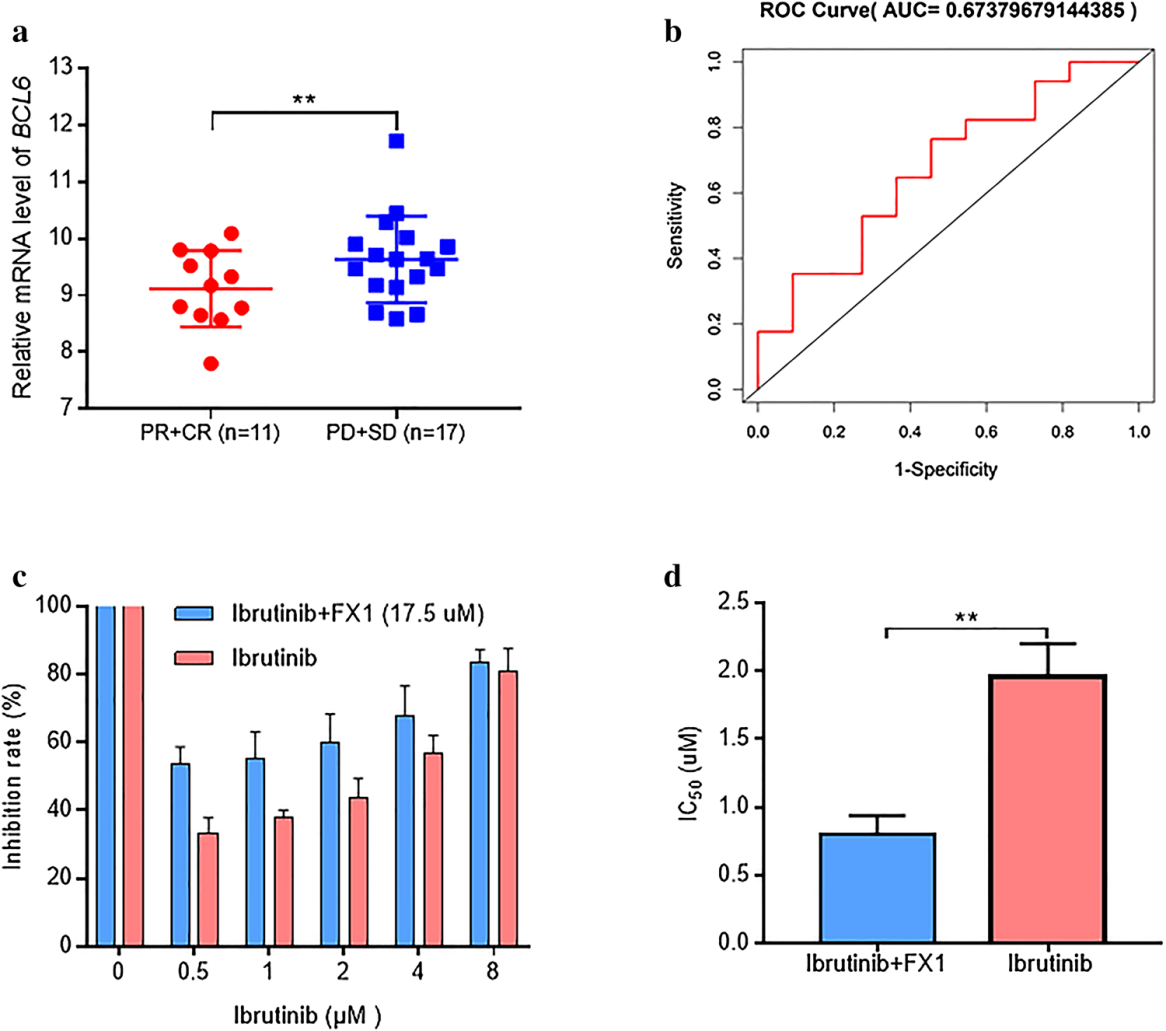
Validation of *BCL6*. **a** *BCL6* was highly expressed in no responsive ABC-DLBCL patients after Ibrutinib treatment. **b** ROC curve for discriminating no response or response through *BCL6* expression. **c** C481S BTK HBL-1 cells were treated with 0–8 uM Ibrutinib or combination with 17.5 uM FX1. **d** MTT-based assessment of the IC_50_ value of Ibrutinib compared with Ibrutinib combining FX1. Values were calculated mean ± SD (*n* = 3, and **P < 0.01)

We got the *BCL6* expression in Ibrutinib pretreated tumor biopsy samples of ABC-DLBCL patients from GSE93984 dataset. The *BCL6* was significantly highly expressed in no responsive than responsive patients (Fig. 6a). The ROC analysis showed that *BCL6* expression can evaluate Ibrutinib effect with an area under ROC curve (AUC) of 0.67 (Fig. 6b). It suggested well confidence of high expression of *BCL6* in no responsive patients after Ibrutinib treatment. We used MTT assay to determine the effect of Ibrutinib with/without FX1 (a *BCL6* inhibitor) on C481S BTK HBL-1 cells. The inhibition rate of C481S BTK HBL-1 cells increased with increasing concentrations of Ibrutinib (Fig. 6c). The IC_50_ value of using Ibrutinib alone was 1.951 ± 0.247 µM, while combining FX1 was 0.800 ± 0.137 µM (Fig. 6d). Thus, FX1 can enhance Ibrutinib sensitivity in C481S BTK HBL-1 cells, which indicated that *BCL6* can decrease Ibrutinib-induced apoptosis in C481S BTK HBL-1 cells.

## Discussion

Even though patients of DLBCL respond sensitively to first-line treatment, approximately 40% of patients still could not benefit from it [4]. Ibrutinib is an inhibitor of BTK, showed obvious efficacy in rel/ref DLBCL, especially ABC subtype [11]. A phase I study of Ibrutinib combined rituximab, ifosfamide, carboplatin, and etoposide (R-ICE) for patients with rel/ref DLBCL reported the high rate of overall response of 90%, including 11 patients achieved complete remission (CR) and 7 patients partial remission (PR) [19]. And another phase III study indicated Ibrutinib and rituximab plus cyclophosphamide, doxorubicin, vincristine, and prednisone (R-CHOP) improved event-free survival (EFS), progression-free survival (PFS), overall survival (OS) in ABC DLBCL patients [20]. Increasing clinical trials investigate Ibrutinib curative effect on DLBCL. We knew that Ibrutinib was approved to be used for several B-cell malignancies in FDA in 2013 [10]. However, the emergence of Ibrutinib resistance has limited its efficacy [21, 22], so it’s important to study the mechanism of Ibrutinib resistance in DLBCL.

In this study, three microarray expression profile datasets were analyzed to further study mechanism of Ibrutinib resistance. First, a total of 237 common DEGs between Ibrutinib sensitive and resistant cell lines were identified through two expression profiles, including 100 upregulated and 137 downregulated genes. Second, GO analysis was performed to study functional roles, including biological process, molecular functions and cellular component. The upregulated DEGs were correlated with transcription and downregulated DEGs were signal transduction and negative regulation of apoptotic process. Third, signal pathway enrichment analysis showed that upregulated DEGs were mainly enriched in cell cycle and downregulated DEGs were mainly enriched in cytokines-based pathways. What is more, PPI network was conducted to illustrate interactions among the DEGs at the protein level, of which, a total of 7 genes-*BCL6*, *IL10*, *IL2RB*, *IRF4*, *CD80*, *PMDR1*, *GZMB*- were selected as hub genes with the threshold of MCC score > 10,000. And through predicting miRNA associated with hub genes, we found that miR-30 family may be related to Ibrutinib resistance in ABC-DLBCL. From the cBioportal database, we found that *BCL6* showed the highest level of genetic alterations among above hub genes. Using another expression profile, we found that *BCL6* highly expressed in no responsive ABC-DLBCL patients, and the AUC of ROC curve was 0.67.

*BCL6*, a transcription repressor, plays an important role of initiation and maintenance of germinal center reactions [23, 24], which has been identified as one of predictors of outcome in several cancers, such as DLBCL and B-cell acute lymphoblastic leukemia (B-ALL) [25, 26]. It was reported that *BCL6* is associated with tyrosine kinase inhibitors (TKI) resistance in Philadelphia chromosome positive (Ph+) ALL and chronic myeloid leukemia (CML) cells [27, 28]. And another study showed overexpression of *BCL6* inhibited the sensitivity of methotrexate in children with B-ALL by promoting *ZEB1* expression [29]. What is more, Julie et al. found association between *BCL6* overexpression and etoposide resistance in DLBCL cell lines [30]. Cardenas et al. reported that *BCL6* expresses in most ABC-DLBCL at a low level [31]. It was interesting that *BCL6* was upregulated in Ibrutinib-resistant ABC-DLBCL cell lines in our study. And *BCL6* had the highest MCC score in Ibrutinib-resistant PPI network and linked with another hub genes. So using another gene profile validated that *BCL6* highly expressed in no responsive ABC-DLBCL patients after Ibrutinib treatment. And in vitro experiment was carried out to validate if *BCL6* inhibitor can enhance the sensitivity of Ibrutinib in C481S BTK HBL-1 cells. FX1, a *BCL6* inhibitor, destroyed the formation of *BCL6* repression complex and suppressed ABC-DLBCL cell lines with IC_50_ of 35 uM [31]. FX1 used in our study was lower than its IC_50_, which can increase sensitivity of Ibrutinb in C481S BTK HBL-1 cells. Thus, our finding that *BCL6* may be involved in drug resistance is consistent with previous studies. What is more, *BCL6* maybe the potential target to improve Ibrutinib sensitivity in C481S BTK HBL-1 cells.

*BCL6* inhibits expression of various target genes via binding gene promoters. *BCL6* not only destroys interactions between T and B cells by *CD80* and PD-L1 but also inhibits B cell differentiation across decreasing expression of *PRDM1* and *IRF4* [32–37]. Above studies are consistent with the upregulation of *BCL6*, and downregulation of other hub genes in our present study. *PRDM1*/*BLIMP1* encodes a transcriptional repressor, which is necessary for differentiation of B cells into plasma cells [38]. Studies reported that *PRDM1* acts as a tumor suppressor gene in ABC-DLBCL in vivo mouse models [39, 40]. As previously described, *PRDM1* is frequently inactivated by genetic alterations, including genetic deletions or mutations or transcriptional repression in ABC-DLBCL [38, 41]. Parekh et al. found that different genetic alterations within *PRDM1* had adverse prognostic factors [42]. And inactivation of *PRDM1* can upregulate expression of *C-MYC* and downregulate expression of *p53* pathway molecule in ABC-DLBCL [42, 43]. These results suggest that inactivation of *PRDM1* is closely linked to development of ABC-DLBCL.

*IRF4* was essential for regulating gene transcription and mitochondrial homeostasis in plasma cells [44]. *IRF4* activates or is repressed by *BCL6*, and co-expresses with *PRPM1* affecting plasma cells development [45–47]. However, most studies found that it does not express *PRDM1* protein though the presence of *IRF4* in ABC-DLBCL, suggesting other regulatory mechanisms between them [40]. Abnormal expression of *IRF4* is linked to several blood malignancies. For example, expression of *IRF4* is related to poor survival outcomes in peripheral T-cell lymphoma and chronic lymphocytic leukemia (CLL) [48, 49]. What is more, studies showed *IRF4* dysregulation is associated with resistance to immunomodulatory compounds in Waldenström’s macroglobulinemia and myeloma [50, 51]. A previous study has reported that Ibrutinib downregulates *IRF4* and consequently synergizes with lenalidomide in killing ABC DLBCL [52]. Another study indicated mutation of *IRF4* may explain the rel/ref phenotype of ABC-DLBCL [5]. Therefore, the role of *IRF4* in ABC-DLBCL need further explore.

Lin et al. identified that upregulation of miR-30 family can directly downregulate *BCL6* in B-lymphocytes and lymphoma cells [53]. Current studies found miR-30 family played a significant role in various tumors. Zhang et al. proved miR-30d could inhibit autophagy thereby promoting cell apoptosis [54]. The higher expression of miRNA-30c had better outcome with tamoxifen treatment in breast cancer [55]. Another investigation found that overexpression of miR-30b and miR-30c have better outcome after TKIs treatment in non-small cell lung cancer [56]. Interestingly, miR-30 family is considered as oncogenic miRNA, too. For instance, Gaziel-Sovran et al. reported that miR-30b and miR-30d promoted invasion of melanoma cells leading to generated *IL10* and reduced immune cells activation and recruitment [57]. Taken together, miR-30 family has complex functions in various cancers. However, the role of miR-30 family in Ibrutinib resistance of ABC-DLBCL has not been reported. Our study showed that miR-30 family may mediate Ibrutinib resistance in ABC-DLBCL, which is worthy of further exploration.

## Conclusion

In summary, the present study has analyzed DEGs based on two microarray expression (GSE138126 and GSE93985). *BCL6* was identified as the core gene for Ibrutinib resistance in ABC-DLBCL. Using another expression profile (GSE93984) showed that *BCL6* highly expressed in no responsive ABC-DLBCL patients after Ibrutinib treatment. Further study found that *BCL6* inhibitor may increase the sensitivity of C481S BTK HBL-1 cells to Ibrutinib therapy. And miRNA target prediction results showed that miR-30 family were involved in Ibrutinib resistance in ABC-DLBCL. And miR-30 family can directly downregulate *BCL6* which was reported before [53]. The *BCL6* maybe a potential target overcoming Ibrutinib resistance in ABC-DLBCL.

## Data Availability

GSE138126, GSE93985, and GSE9394 were downloaded from the Gene Expression Omnibus (GEO) database.

## References

[1] Bachy E, Salles G (2015). Treatment approach to newly diagnosed diffuse large B-cell lymphoma. Semin Hematol.

[2] Swerdlow SH, Campo E, Pileri SA, Harris NL, Stein H, Siebert R (2016). The 2016 revision of the World Health Organization classification of lymphoid neoplasms. Blood.

[3] Lenz G, Wright G, Dave SS, Xiao W, Powell J, Zhao H (2008). Stromal gene signatures in large-B-Cell Lymphomas. N Engl J Med.

[4] Li S, Young KH, Medeiros LJ (2018). Diffuse large B-cell lymphoma. Pathology.

[5] Mareschal S, Dubois S, Viailly PJ, Bertrand P, Bohers E, Maingonnat C (2016). Whole exome sequencing of relapsed/refractory patients expands the repertoire of somatic mutations in diffuse large B-cell lymphoma. Genes Chromosomes Cancer.

[6] Morin RD, Assouline S, Alcaide M, Mohajeri A, Johnston RL, Chong L (2016). Genetic landscapes of relapsed and refractory diffuse large B-cell lymphomas. Clin Cancer Res.

[7] Jørgensen LK, Poulsen MØ, Laursen MB, Marques SC, Johnsen HE, Bøgsted M (2015). MicroRNAs as novel biomarkers in diffuse large B-cell lymphoma—a systematic review. Dan Med J.

[8] Garzon R, Calin GA, Croce CM (2009). MicroRNAs in cancer. Annu Rev Med.

[9] Ting CY, Liew SM, Price A, Gan GG, Bee-Lan Ong D, Tan SY (2019). Clinical significance of aberrant microRNAs expression in predicting disease relapse/refractoriness to treatment in diffuse large B-cell lymphoma: a meta-analysis. Crit Rev Oncol Hematol.

[10] Burger JA, Buggy JJ (2013). Bruton tyrosine kinase inhibitor ibrutinib (PCI-32765). Leuk Lymphoma.

[11] Wilson WH, Young RM, Schmitz R, Yang Y, Pittaluga S, Wright G (2015). Targeting B cell receptor signaling with ibrutinib in diffuse large B cell lymphoma. Nat Med.

[12] Edgar R, Domrachev M, Lash AE (2002). Gene expression omnibus: NCBI gene expression and hybridization array data repository. Nucleic Acids Res.

[13] Barrett T, Wilhite SE, Ledoux P, Evangelista C, Kim IF, Tomashevsky M (2013). NCBI GEO: archive for functional genomics data sets–update. Nucleic Acids Res.

[14] da Huang W, Sherman BT, Lempicki RA (2009). Systematic and integrative analysis of large gene lists using DAVID bioinformatics resources. Nat Protoc.

[15] Szklarczyk D, Gable AL, Lyon D, Junge A, Wyder S, Huerta-Cepas J (2019). STRING v11: protein-protein association networks with increased coverage, supporting functional discovery in genome-wide experimental datasets. Nucleic Acids Res.

[16] Almeida D, Azevedo V, Silva A, Baumbach J (2016). PetriScape—a plugin for discrete Petri net simulations in Cytoscape. Journal of integrative bioinformatics.

[17] Gao J, Aksoy BA, Dogrusoz U, Dresdner G, Gross B, Sumer SO (2013). Integrative analysis of complex cancer genomics and clinical profiles using the cBioPortal. Sci Signal.

[18] Wong N, Wang X (2015). miRDB: an online resource for microRNA target prediction and functional annotations. Nucleic Acids Res.

[19] Sauter CS, Matasar MJ, Schoder H, Devlin SM, Drullinsky P, Gerecitano J (2018). A phase 1 study of ibrutinib in combination with R-ICE in patients with relapsed or primary refractory DLBCL. Blood.

[20] Younes A, Sehn LH, Johnson P, Zinzani PL, Hong X, Zhu J (2019). Randomized phase III trial of ibrutinib and rituximab plus cyclophosphamide, doxorubicin, vincristine, and prednisone in non-germinal center B-cell diffuse large B-cell lymphoma. J Clin Oncol.

[21] Dreyling M, Jurczak W, Jerkeman M, Silva RS, Rusconi C, Trneny M (2016). Ibrutinib versus temsirolimus in patients with relapsed or refractory mantle-cell lymphoma: an international, randomised, open-label, phase 3 study. Lancet (London, England).

[22] Maddocks KJ, Ruppert AS, Lozanski G, Heerema NA, Zhao W, Abruzzo L (2015). Etiology of ibrutinib therapy discontinuation and outcomes in patients with chronic lymphocytic leukemia. JAMA Oncol.

[23] Basso K, Dalla-Favera R (2010). BCL6: master regulator of the germinal center reaction and key oncogene in B cell lymphomagenesis. Adv Immunol.

[24] Hatzi K, Melnick A (2014). Breaking bad in the germinal center: how deregulation of BCL6 contributes to lymphomagenesis. Trends Mol Med.

[25] Lossos IS, Jones CD, Warnke R, Natkunam Y, Kaizer H, Zehnder JL (2001). Expression of a single gene, BCL-6, strongly predicts survival in patients with diffuse large B-cell lymphoma. Blood.

[26] Hurtz C, Chan LN, Geng H, Ballabio E, Xiao G, Deb G (2019). Rationale for targeting BCL6 in MLL-rearranged acute lymphoblastic leukemia. Genes Dev.

[27] Eskandari S, Yazdanparast R (2017). Bcl6 gene-silencing facilitates PMA-induced megakaryocyte differentiation in K562 cells. J Cell Commun Signal.

[28] Duy C, Hurtz C, Shojaee S, Cerchietti L, Geng H, Swaminathan S (2011). BCL6 enables Ph+ acute lymphoblastic leukaemia cells to survive BCR-ABL1 kinase inhibition. Nature.

[29] Wu HB, Lv WF, Wang YX, Li YY, Guo W (2018). BCL6 promotes the methotrexate-resistance by upregulating ZEB1 expression in children with acute B lymphocytic leukemia. Eur Rev Med Pharmacol Sci.

[30] Devin J, Kassambara A, Bruyer A, Moreaux J, Bret C (2019). Phenotypic characterization of diffuse large B-cell lymphoma cells and prognostic impact. J Clin Med.

[31] Cardenas MG, Yu W, Beguelin W, Teater MR, Geng H, Goldstein RL (2016). Rationally designed BCL6 inhibitors target activated B cell diffuse large B cell lymphoma. J Clin Invest.

[32] Parekh S, Polo JM, Shaknovich R, Juszczynski P, Lev P, Ranuncolo SM (2007). BCL6 programs lymphoma cells for survival and differentiation through distinct biochemical mechanisms. Blood.

[33] Shaffer AL, Yu X, He YS, Boldrick J, Chan EP, Staudt LM (2000). BCL-6 represses genes that function in lymphocyte differentiation, inflammation, and cell cycle control. Immunity.

[34] Tunyaplin C, Shaffer AL, Angelin-Duclos CD, Yu X, Staudt LM, Calame KL (2004). Direct repression of prdm1 by Bcl-6 inhibits plasmacytic differentiation. J Immunol (Baltimore, Md: 1950).

[35] Niu H, Cattoretti G, Dalla-Favera R (2003). BCL6 controls the expression of the B7–1/CD80 costimulatory receptor in germinal center B cells. J Exp Med.

[36] Basso K, Saito M, Sumazin P, Margolin AA, Wang K, Lim WK (2010). Integrated biochemical and computational approach identifies BCL6 direct target genes controlling multiple pathways in normal germinal center B cells. Blood.

[37] Basso K, Dalla-Favera R (2012). Roles of BCL6 in normal and transformed germinal center B cells. Immunol Rev.

[38] Shapiro-Shelef M, Lin KI, McHeyzer-Williams LJ, Liao J, McHeyzer-Williams MG, Calame K (2003). Blimp-1 is required for the formation of immunoglobulin secreting plasma cells and pre-plasma memory B cells. Immunity.

[39] Calado DP, Zhang B, Srinivasan L, Sasaki Y, Seagal J, Unitt C (2010). Constitutive canonical NF-κB activation cooperates with disruption of BLIMP1 in the pathogenesis of activated B cell-like diffuse large cell lymphoma. Cancer Cell.

[40] Mandelbaum J, Bhagat G, Tang H, Mo TW, Brahmachary M, Shen Q (2010). BLIMP1 is a tumor suppressor gene frequently disrupted in activated B cell-like diffuse large B cell lymphoma. Cancer Cell.

[41] Tam W, Gomez M, Chadburn A, Lee JW, Chan WC, Knowles DM (2006). Mutational analysis of PRDM1 indicates a tumor-suppressor role in diffuse large B-cell lymphomas. Blood.

[42] Xia Y, Xu-Monette ZY, Tzankov A, Li X, Manyam GC, Murty V (2017). Loss of PRDM1/BLIMP-1 function contributes to poor prognosis of activated B-cell-like diffuse large B-cell lymphoma. Leukemia.

[43] Zhang XY, Ma ZP, Cui WL, Pang XL, Chen R, Wang L (2018). Impact of PRDM1 gene inactivation on C-MYC regulation in diffuse large B-cell lymphoma. Chin J Pathol.

[44] Low MSY, Brodie EJ, Fedele PL, Liao Y, Grigoriadis G, Strasser A (2019). IRF4 activity is required in established plasma cells to regulate gene transcription and mitochondrial homeostasis. Cell Rep.

[45] Angelin-Duclos C, Cattoretti G, Lin KI, Calame K (2000). Commitment of B lymphocytes to a plasma cell fate is associated with Blimp-1 expression in vivo. J Immunol (Baltimore, Md: 1950).

[46] Shapiro-Shelef M, Calame K (2005). Regulation of plasma-cell development. Nat Rev Immunol.

[47] Klein U, Dalla-Favera R (2008). Germinal centres: role in B-cell physiology and malignancy. Nat Rev Immunol.

[48] Heo MH, Park HY, Ko YH, Kim WS, Kim SJ (2017). IRF4/MUM1 expression is associated with poor survival outcomes in patients with peripheral T-cell lymphoma. J Cancer.

[49] Chang CC, Lorek J, Sabath DE, Li Y, Chitambar CR, Logan B (2002). Expression of MUM1/IRF4 correlates with clinical outcome in patients with B-cell chronic lymphocytic leukemia. Blood.

[50] Zhu YX, Shi CX, Bruins LA, Wang X, Riggs DL, Porter B (2019). Identification of lenalidomide resistance pathways in myeloma and targeted resensitization using cereblon replacement, inhibition of STAT3 or targeting of IRF4. Blood Cancer J.

[51] Bertrand E, Jouy N, Manier S, Guillemette F, Guidez S, Eileen B (2017). Role of IRF4 in resistance to immunomodulatory (IMid) compounds® in Waldenström’s macroglobulinemia. Onco Targets Ther.

[52] Yang Y, Shaffer AL, Emre NT (2012). Exploiting synthetic lethality for the therapy of ABC diffuse large B cell lymphoma. Cancer Cell.

[53] Lin J, Lwin T, Zhao JJ (2011). Follicular dendritic cell-induced microRNA-mediated upregulation of PRDM1 and downregulation of BCL-6 in non-Hodgkin’s B-cell lymphomas. Leukemia.

[54] Zhang R, Xu J, Zhao J, Bai J (2017). Mir-30d suppresses cell proliferation of colon cancer cells by inhibiting cell autophagy and promoting cell apoptosis. Tumour Biol.

[55] Rodríguez-González FG, Sieuwerts AM, Smid M, Look MP, Meijer-van Gelder ME, de Weerd V (2011). MicroRNA-30c expression level is an independent predictor of clinical benefit of endocrine therapy in advanced estrogen receptor positive breast cancer. Breast Cancer Res Treat.

[56] Gu YF, Zhang H, Su D, Mo ML, Song P, Zhang F (2013). miR-30b and miR-30c expression predicted response to tyrosine kinase inhibitors as first line treatment in non-small cell lung cancer. Chin Med J.

[57] Gaziel-Sovran A, Segura MF, Di Micco R, Collins MK, Hanniford D, Vega-Saenz de Miera E (2011). miR-30b/30d regulation of GalNAc transferases enhances invasion and immunosuppression during metastasis. Cancer Cell.

